# Meta‐Analysis of Solution‐Focused Brief Therapy Research Conducted in Iran: Does Outcome Type, Intervention Modality, or Delivery Format Make a Difference?

**DOI:** 10.1111/jmft.12759

**Published:** 2024-12-29

**Authors:** Adam S. Froerer, Mustafa Bolghan‐Abadi, Meiyan Chen, Anao Zhang

**Affiliations:** ^1^ The Solution Focused Universe Fort Worth Texas USA; ^2^ Department of Educational Sciences, Faculty of Literature and Humanities Hakim Sabzevari University Sabzevar Iran; ^3^ Steve Hicks School of Social Work The University of Texas at Austin Austin Texas USA; ^4^ School of Social Work University of Michigan Ann Arbor Michigan USA

## Abstract

This meta‐analysis synthesized randomized and non‐randomized controlled trials of solution‐focused brief therapy (SFBT) in Iranian populations, examining the effectiveness and the applications of SFBT in all Iranian settings and evaluated if the various outcomes being studied, research intervention modality, or therapy delivery format made statistical differences in outcomes. A comprehensive search strategy across three Iranian and four US databases, one Iranian conference website, and three professional websites resulted in the inclusion of 34 included studies for meta‐analytic analysis. The outcomes reported an overall large significant treatment effect size (*d* = 0.977, 95% CI: 0.591, 1.37, *p* < 0.001), with subgroup analysis revealing large effects for interventions for *psychosocial* and *mental health* (*g* = 1.24, *p* < 0.001) and *self‐wellness* (*g* = 0.903, *p* = 0.00274). SFBT was also shown to be effective in group settings (*d* = 1.04, *p* < 0.001). Results indicated that SFBT was an effective intervention for Iranian populations.

## 1 Introduction

Solution‐Focused Brief Therapy (SFBT) is a psychotherapeutic approach that is backed by a significant body of literature supporting its effectiveness (Kim et al. 2019). SFBT takes a progressive perspective that persons seeking therapy are competent, capable, and have the resources necessary to make desired changes in their lives (Connie and Froerer 2023). SFBT clinicians use a language‐based approach to co‐construct hope‐filled and strengths‐based conversations that are in line with the client's desired outcome for therapy and to create meaningful and lasting change (Connie 2018; De Shazer et al. 2007). SFBT values the autonomy and agency of clients while focusing on the changes the client would like to achieve (Connie and Froerer 2023).

Because SFBT is language‐oriented, the specific words clients use are significant and the way clinicians utilize these words to formulate their next questions is also very important (Jordan, Froerer, and Bavelas 2013). Meaningful use of language is the foundation of SFBT and is often the focus of evaluating the effectiveness of SFBT. The solution‐focused approach is growing in popularity and is being utilized around the world (Park et al. 2024, González Suitt et al. 2019).

The respectful, autonomy‐honoring approach of SFBT helps to make it broadly applicable to a wide range of clients with various presenting problems (Franklin et al. 2022; Schade, Torres, and Beyebach 2011; Zhang et al. 2017). There is growing evidence that SFBT, although developed in the United States, is broadly applicable to populations around the globe (Beyebach et al. 2021). Although there is significant supportive research available for SFBT, much of the research has been conducted in Western countries and with Western populations. Research has begun to investigate the effectiveness of SFBT in a wide variety of global locations. Iran is one such country where more and more research is being conducted about SFBT and its effectiveness.

## 2 Literature Review

There have been several meta‐analyses conducted around the globe that support the effectiveness of SFBT. Some countries where SFBT meta‐analyses have been conducted include China (Kim et al. 2015), Korea (Park 2014), The Netherlands (Stams et al. 2006), Taiwan (Gong and Hsu 2015), Turkey (Karababa 2023), and the United States (Franklin et al. 2022; Kim 2008). These multiple meta‐analyses have shown that SFBT is effective with children, adolescents, and adults. The studies have resulted in small, medium, and large effects with changing personal behavior, internalizing and externalizing behaviors, the impacts of medical conditions, parenting concerns, school‐related concerns, and mental health concerns. The body of SFBT empirically supported research is becoming popular and is very convincing.

Despite the growing meta‐analytic support for SFBT, we must refrain from over‐generalizing the findings and make the positive results applicable worldwide without corresponding research to support this generalization. Many of the meta‐analyses cited above were conducted to specifically look at the impact of SFBT within a given country or a specific delivery setting (i.e., medical settings or schools). This narrow scope can give us important information and add support to the overall literature base but should be interpreted with caution and should not be broadly interpreted to insinuate the global effectiveness of the approach. Additionally, given that the SFBT research literature is still a relatively young field compared with other psychotherapeutic approaches like cognitive behavioral therapy, many existing meta‐analytic studies are limited in their abilities to evaluate some important moderators that are related to the heterogeneity in SFBT's treatment outcomes.

For example, few Meta‐analytic studies were unable to evaluate the moderating effect of the experimental design type in relation to SFBT's treatment effect size, mainly because there was not sufficient study for the comparison (Zhang et al. 2017). Some evidence in the SFBT school literature suggests that non‐individual‐delivered SFBT may have a larger treatment effect than individual‐based SFBT (Franklin et al. 2022). Given the current state of the SFBT literature, it is important to continue the evaluation of the SFBT literature with an ever‐expanding population.

Iran is a country where more and more research is being conducted about SFBT, with some of the studies published in English and others published in Farsi/Persian. Beyebach and colleagues (2021) highlight that looking at the effectiveness among non‐Western, non‐wealthy, and nondemocratic countries, such as Iran, could be useful in understanding the potentially different impacts of SFBT. Beyebach et al. suggest that Iran is one of the most prolific locations for newly published SFBT research from non‐Western countries. Despite the global support of meta‐analyses and the growing body of SFBT research from Iran specifically, as far as we are aware, there has never been a published meta‐analysis of the Iranian studies. This is a significant gap in the current SFBT literature.

As a beginning stop‐gap for this literature void, one scoping review of Iranian literature was conducted by Reddy et al. (2022). Reddy and colleagues reported that SFBT was predominantly conducted in urban areas (i.e. Tehran [35.1%], Isfahan [27.0%], and Mashhad [25.7%]) and with individuals in Iran (74.0% of the time), although SFBT had been utilized with couples as well (26.0% of the time). About 57% of study participants were adults; however, children (24.7%) and adolescents (14.3%) were also included in the research. Finally, in the same study, Reddy et al. (2022) reported that about 50.6% of the Iranian studies looked at the impact of SFBT on internalizing behavior problems, and another 48.1% of studies looked at dyadic satisfaction and conflict. Although this scoping review helps provide a beginning understanding of the SFBT literature in Iran, it does not empirically evaluate the effectiveness of the approach with an Iranian population. This study used a systematic review and meta‐analysis approach to increase rigor. This study is aimed at understanding if SFBT is effective with Iranian populations and seeks to understand if outcome type (area of treatment focus), study design (randomized controlled trial [RCT] or non‐RCT), or intervention modality (i.e., individual, couple, or group) makes a difference in research outcomes.

## 3 Methods

### 3.1 Selection Criteria

The study objective was to examine the effectiveness and the applications of SFBT in all Iranian settings and to evaluate if the various outcomes being studied, research intervention modality or therapy delivery format made a difference. In this paper, we focused on papers published in English literature. Our search included published manuscripts, unpublished manuscripts that are available online, dissertations, and other gray literature, such as government reports, white papers, or conference proceedings.

### 3.2 Search Strategy

We used three search strategies to create the initial list of potential studies for this meta‐analysis. First, we conducted three electronic searches with Iranian databases (Magiran, SID, and Noormags). These are the three most commonly used databases in Iran for Social Science publications. We used the following search terms within each of the Iranian Databases, (SFBT or solution* or solution focused or solution‐focused) AND (effect* or effic*) AND Iran (solution oriented or solution‐oriented) AND (effect* or effic*) AND Iran (circuit solution) AND (effect* or effic*) AND Iran. These terms were used due to translation variability and to cast the widest search possible. We searched each of these databases for any published articles, regardless of publication date; again, the goal was to assess all of the current available literature. We completed a similar search, with the same search terms, in seven U.S. electronic databases: Child Development & Adolescent Studies, CINAHL Complete, Family Studies Database, PsycINFO, Social Sciences Abstract, the Cochrane Library, and MEDLINE. We used the EBSCO host for cross‐database search and completed all our searches on November 1, 2023.

Our second search strategy was to search for conference and presentation workshops (gray literature) using the Persian website Civilica (https://en.civilica.com). It was hoped that we would be able to broaden the included papers and reduce publication bias with this additional search. It should be noted that we decided not to include any of the 33 items identified within this search because all these identified studies did not meet the design requirement (i.e., being a controlled trial) or did not have the necessary statistical information for effect sizes for inclusion.

Finally, we manually reviewed and searched major internet sources for SFBT literature to include. We reviewed literature lists from The Solution Focused Universe's searchable database at https://solutionfocusedbrieftherapy.com/searchable-database/, The Institute for Solution‐Focused Therapy at https://solutionfocused.net/, and The Solution‐Focused Brief Therapy Association's list at http://www.sfbta.org. There were no new items found in this search beyond the articles previously located by the database searches.

### 3.3 Study Eligibility

To be eligible for inclusion, a study needed to include SFBT as a treatment option in a randomized controlled trial (RCT) or a quasi‐experimental control study with comparison group(s). The included studies also needed to be conducted in Iran with populations residing in Iran. It should be noted that some studies included non‐Iranian citizens within their sample populations. Given that this study is the first meta‐analysis conducted with Iranian populations, we did not limit the purpose or scope of the presenting problems or the purpose of the original studies. Given the exploratory nature of this study, we also chose not to use the age of participants as an exclusionary criterion. Because the SFBT techniques used with different aged participants do not change and this language‐based approach would be customized for each individual participant, it was determined that all results should be considered together. Post‐hoc analysis was conducted to determine if age impacted outcomes. This resulted in a wide variety of populations included in the analysis. Some of the resulting populations were adolescents, school‐aged children, adults with medical conditions, couples, adults with general mental health concerns (i.e., depression, anxiety, low self‐esteem), and domestic violence. It should also be noted that when couples were included, data from each partner was included when possible.

Studies were excluded if they were not conducted in Iran or if SFBT was not used as a treatment approach within the study. The SFBT components (techniques or skills) mentioned specifically in the protocols for the original studies were noted and considered as part of the analysis. To determine if a study qualified as a legitimate SFBT intervention, the study needed to (1) clearly state specific SFBT techniques or skills used as part of the protocol that was included in an SFBT treatment manual, or (2) clearly state which components of SFBT they used that the researchers could then verify were consistent with components that could be found within SFBT treatment manuals. Most studies listed the week‐by‐week SFBT components used within the study protocol.

### 3.4 Study Screening and Data Extraction

**Figure 1:**
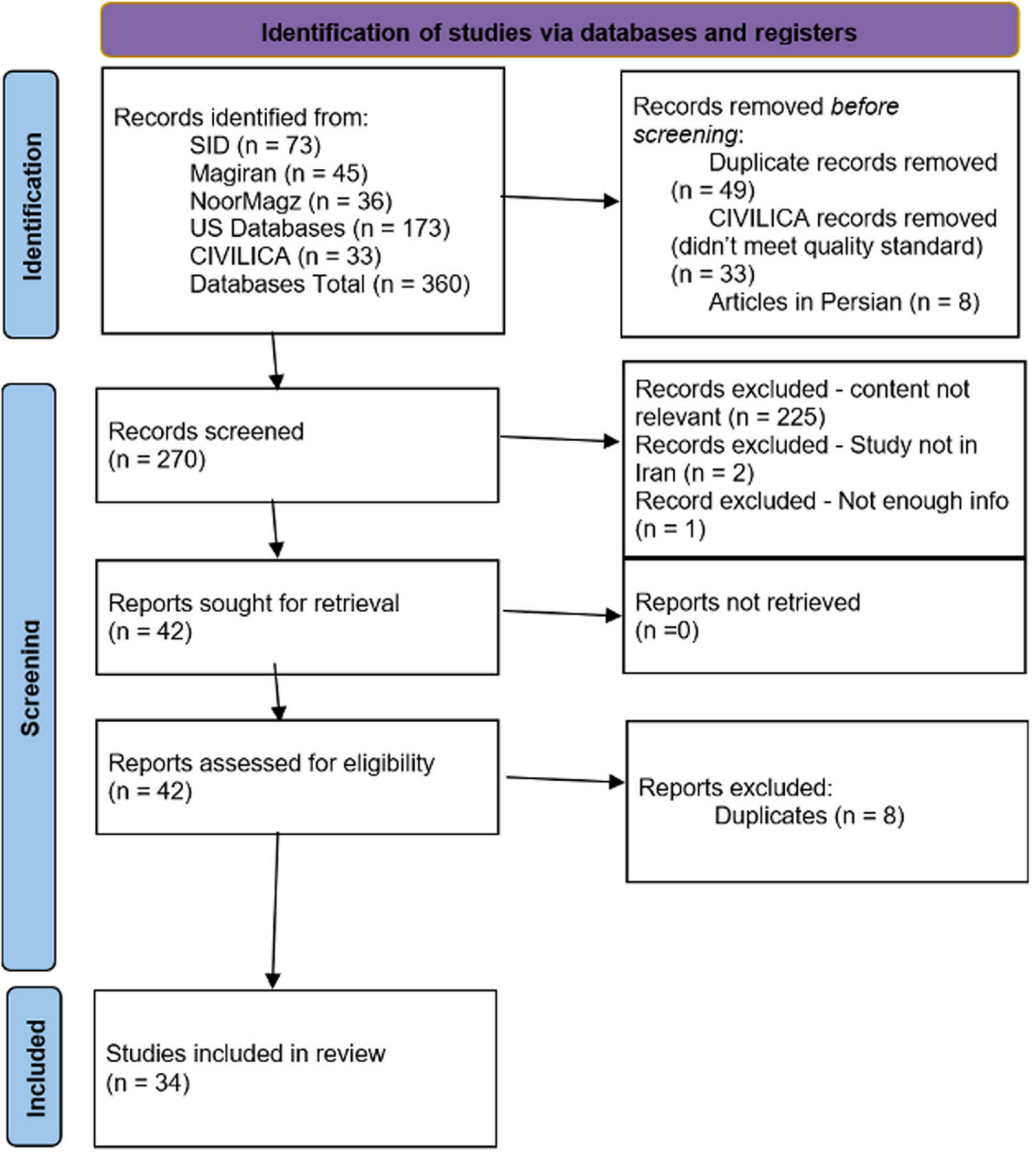
PRISMA 2020 flow diagram. *Source:* Page MJ, McKenzie JE, Bossuyt PM, Boutron I, Hoffmann TC, Mulrow CD, et al. The PRISMA 2020 Statement: An Updated Guideline for Reporting Systematic Reviews. *BMJ* 2021;372:n71. doi:10.1136/bmj.n71

Figure 1 displays the PRISMA 2020 flow diagram of search steps and the exclusion process. The original search terms resulted in the identification of 360 total studies, of which 154 studies were pulled from the Iranian databases and 173 studies from the US databases. As described above, 33 gray literature items identified through CIVILICA were excluded as they did not meet the criteria for inclusion due to design or statistical information. After removing these items along with 49 duplicated items and 8 papers that were published in Persian, 270 abstracts were screened by the authors for relevance to the study. In total, 228 studies were excluded due to their irrelevance to SFBT or not being conducted in Iran. One study was excluded because it did not have the necessary statistical data required for the analysis. After the abstract review, all 42 papers were reviewed in totality and 8 additional papers were excluded due to duplications. All remaining papers were screened for statistical eligibility, meaning they contained means and standard deviation scores for both treatment and comparison group(s). Studies that included results from independent *t*‐tests, as well as two group analyses of variance and binary logistical regression were also included, though all outcomes of interest were continuous outcomes. Thirty‐four papers remained for coding, data analysis, and reporting results.

The main data extractors (A.F. and M.B.) are experienced SFBT clinicians and researchers. They extracted the data from each of the included studies using a coding sheet developed specifically for this project (available as a supplement to this paper). The two extractors conducted the extraction of all studies independently without knowing the others' coding, and the results were compared later on. If questions arose during the extraction process a third researcher (A.Z.) was consulted for clarification and input. The coding sheet extracted demographic and diagnostic data about participants (where possible), details about the study design and intervention parameters (number of sessions, duration of each session, etc.), specific SFBT interventions utilized within the research process, details about the person providing the intervention, and statistical data, i.e., means and standard deviations of outcomes, at baseline, immediately following intervention, and at follow‐up intervals for control and comparison groups. Efforts were made to contact the authors when there was missing data.

### 3.5 Risk of Bias

The Revised Cochrane Risk‐of‐Bias tool 2nd version and the Non‐Randomized Studies of the Effects of Interventions (ROBINS‐I) were used to assess the risk of bias for both randomized controlled trials (31/34) and controlled trials without random assignment (3/34) in this review, respectively. A study's quality was rated by two independent reviewers and compared for consistency (with a 98% rate of consistency). Disagreements were discussed by the two reviewers first to reach an agreement and, if unsuccessful, being determined by a third reviewer in the study team.

### 3.6 Meta‐Analytic Procedures

Data analysis proceeded in four phases, and all analyses were conducted in R software. In phase one, descriptive statistics describing study characteristics were conducted. Then, researchers calculated small sample size corrected effect size estimates for each study to determine the magnitude of the treatment effect. Because all research outcomes were continuous in nature, we calculated the between‐groups standardized mean difference (SMD), i.e., Hedges' *g* (Cooper, Hedges, and Valentine 2019). In accordance with the best practice, researchers further adjusted the *g* statistic with a small sample size correction to obtain an unbiased estimate of treatment effect size, denoted as *d* in this study (Cooper, Hedges, and Valentine 2019). To synthesize effect size estimates across the included studies, this study used meta‐regression with robust variance estimation (RVE) (Hedges, Tipton, and Johnson 2010; Tipton and Pustejovsky 2015). Meta‐regression with RVE was chosen because it effectively uses an intercept‐only model to give an overall average of treatment effect sizes across studies. Not only does this analytical approach handle dependent effect sizes (i.e., when more than one effect size estimate is reported within one study and all reported effect size estimates are included), but it also produces robust statistical inference regardless of a model's variance modeling strategy, i.e., the selection between a fixed‐ versus a random‐effects modeling strategy (Hedges, Tipton, and Johnson 2010). Given that our analysis included effect sizes from multiple measures of the same construct (i.e., correlated effects) and multiple effect sizes within/from the same study (i.e., hierarchical effects), we proceeded with an expanded working model of RVE, i.e., the correlated and hierarchical effects model (CHE) (https://events.brown.edu/live/files/594-pustejovsky-tipton-2021pdf). The CHE model combines the desirable features of both the correlated and hierarchical effects models, allowing for both between‐study heterogeneity and within‐study heterogeneity in true effect sizes as well as the correlation between effect size estimates within each study (https://events.brown.edu/live/files/594-pustejovsky-tipton-2021pdf). In addition, meta‐regression with RVE utilizes list‐wise deletion to accommodate missing data. Finally, researchers also planned subgroup analyses and univariate moderator analyses based on outcome categories, which were grouped post‐hoc‐based outcome measures. The “metaphor” and “robumeta” packages of R Statistical Software (version 4.2.2) were used for all data analyses.

## 4 Results

### 4.1 Study Characteristics

The 34 trials included 156 effect size estimates and a total sample size of 1542 participants in the SFBT intervention. Trials were published between 2012 and 2022, including 6 published between 2012 and 2015, 16 published between 2016 and 2019, and 12 published between 2020 and 2022. Participants' average age ranged from 10.5 to 57.73 years. In the trials that reported patients' sex (*n* = 29), 72.11% of the patients were female (*n* = 1112). Except three studies, every study was a randomized controlled trial (RCT), and three of those were non‐randomized controlled trials. In the present review, among the twenty‐five studies that reported frequency of intervention, one study adopted a twice‐a‐week intervention schedule, another study utilized a once‐every‐other‐week frequency, and the majority of the investigations (23/25) implemented a weekly intervention regimen. Over 90% of the studies (32/34) used group‐based intervention as the primary method of intervention, while two studies used individual‐based intervention as their intervention strategy. Most clinical trials (29/34) reported individual‐level outcomes, leaving five studies whose respondents reported couple‐level outcomes. A table describing the study characteristics is attached as a supplement.

### 4.2 Publication Bias

**Figure 2:**
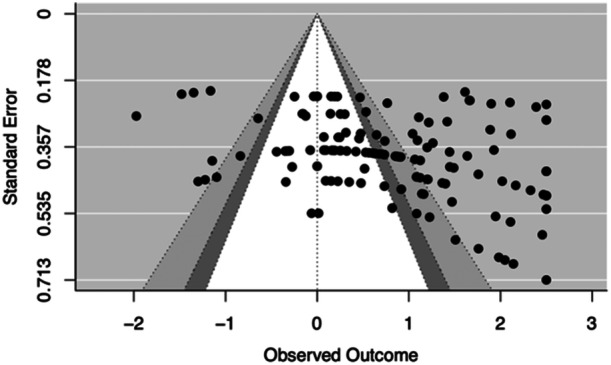
Funnel plot for publication bias.

Both the funnel plot and the Vevea and Woods (2005) sensitivity analysis with a weight‐function model were used to evaluate publication bias. The funnel plot illustrating publication bias (Figure 2) exhibited an absence of data points with lower outcomes and standard errors, implying the potential presence of publication bias in the current study. Nonetheless, the application of Vevea and Woods' weight model, which is a statistical model to evaluate publication bias, did not substantiate the existence of publication bias. The observed overall treatment effect size was found to be statistically nonsignificant when compared to the theoretical overall treatment effect size, assuming the funnel plot is symmetric. Given the result of the statistical modeling on publication bias, we do not consider publication as a concern in this study.

### 4.3 Risk of Bias

In general, studies included in this review identified a low risk of bias in addressing missing outcome data (32/34), appropriate reporting in the measurement of the outcome (34/34), and concern in the selection of the reported results (33/34). However, twenty‐one randomized controlled trials reported a moderate risk of bias arising from the randomization process, and three non‐randomized controlled trials indicated a moderate risk of bias in the selection of participants into the study. Besides, moderate (3/34) and high risk of bias (3/34) due to deviations from the intended interventions were observed in included studies. In controlled trials without random assignment, three studies had a moderate risk of bias in the selection of participants for the study. Taken together, included studies reported a relatively low risk of bias. The risk of bias table is also included as an attachment.

### 4.4 Meta‐Analytic Results

Across the 34 studies (containing 156 effect sizes) included in this meta‐analysis, the pooled treatment effect size of SFBT was *d* = 0.977, 95% CI [0.591, 1.37], *p* < 0.001, indicating a statistically significant large treatment effect of SFBT interventions. In comparison to the control condition, participants who received interventions were, on average, 0.977 standard deviations better (improved) in all the outcomes. Results were evaluated based on outcome subgroups. These subgroups were categorized by the researchers based on the research criteria of the original studies. The five outcome subgroups included, 1) psychological and mental health (i.e., distress, anxiety, depression, etc.), 2) social/relational (i.e., marital satisfaction, communication, intimacy, etc.), 3) self‐wellness (i.e., assertiveness, self‐efficacy, self‐confidence, etc.), 4) general wellness (i.e., physical symptoms, quality of life, general health, etc.), and 5) personal traits/characteristics (i.e., problem‐solving, organization, cooperativeness, etc.). Subgroup analysis revealed that SFBT interventions for psychosocial and mental health, *g* = 1.24, 95% CI [0.816, 1.67], *p* < 0.001, and self‐wellness, *g* = 0.903, 95% CI [0.374, 1.43], *p* = 0.00274, reported overall statistically significant treatment effects, respectively. Correspondingly, SFBT interventions for participants' social/relationship outcomes, *g* = 0.613, 95% CI [‐0.037, 1.26], *p* = 0.0632, general wellness outcomes, *g* = 0.784, 95% CI [‐0.158, 1.73], *p* = 0.092, and personal traits/characteristics outcomes, *g* = 0.825, 95% CI [0.008, 1.64], *p* = 0.049, were not statistically significant.

Aside from subgroup analyses based on outcome categories, statistically significant treatment effects were identified among additional subgroups. First, for study design subgroup analyses, an overall significant treatment effect was observed only among randomized controlled trials, *d* = 0.95, 95% CI [0.524, 1.38], *p* < 0.001. Second, in intervention modality subgroup analyses, treatment effects were statistically significant for group‐based interventions only, *d* = 1.04, 95% CI [0.629, 1.44], *p* < 0.001. Third, in respondent‐type subgroup analyses, only the individual group reported overall statistically significant treatment effects, *d* = 0.931, 95% CI [0.567, 1.3], *p* < 0.001. Finally, treatment effects were found statistically significant for both follow‐up groups, *d* = 0.94, 95% CI [0.527, 1.35], *p* < 0.001, and test after intervention group *d* = 1.18, 95% CI [0.604, 1.76], *p* = 0.00126, in follow‐up subgroup analyses (Tables 1).

**Table 1:** Overall and subgroup meta‐analysis.

Variables	Estimate	SE^a^	K/N^b^	95% CI^c^	dfs^d^	*p*
Overall effect	0.977	0.191	34/156	[0.591, 1.37]	32.9	< 0.001***
Outcome type						
Psychological and mental health	1.24	0.205	25/62	[0.816, 1.67]	23.9	< 0.001***
Social/relationship	0.613	0.301	14/26	[−0.037, 1.26]	13	0.0632
Self‐wellness	0.903	0.245	14/39	[0.374, 1.43]	12.9	0.00274**
General wellness	0.784	0.416	10/22	[−0.158, 1.73]	8.99	0.0923
Personal traits/characteristics	0.825	0.189	3/7	[0.008, 1.64]	1.99	0.0492
Study design						
Randomized controlled trial	0.95	0.208	31/145	[0.524, 1.38]	29.9	< 0.001***
Non‐randomized controlled trial	1.33	0.129	3/11	[0.757, 1.9]	1.93	0.0104
Intervention modality						
Group‐based intervention	1.04	0.199	32/147	[0.629, 1.44]	30.9	< 0.001***
Individual‐based intervention	0.081	0.020	2/9	[−0.169, 0.331]	1	0.152
Respondent type						
Individual	0.931	0.178	29/133	[0.567, 1.3]	27.9	< 0.001***
Couple	1.23	0.836	5/23	[−1.09, 3.55]	4	0.214
Follow‐up						
Test after intervention	0.94	0.203	32/119	[0.527, 1.35]	30.9	< 0.001***
Follow‐up test	1.18	0.256	10/37	[0.604, 1.76]	8.97	0.00126**

^a^ SE = standard error.

^b^ *K* = number of studies, *N* = number of effect size estimates.

^c^ CI = confidence Interval.

^d^ dfs, degrees of freedom. If dfs < 4, a lower *p*‐value (*p* < 0.01) should be used as a threshold for statistical significance.

*** *p* < 0.001

** *p* < 0.01, *p < 0.05.

### 4.5 Moderator Analysis

Given the substantial degree of heterogeneity, *Q* (155) = 1492.413, *p* < 0.0001, among effect size estimates, moderator analyses (Table 2) were carried out to determine if any moderator could be used to account for the variations in effect sizes. Moderators examined included (1) outcome types (psychological mental health, social relation, self‐wellness, general health, personal traits/characteristics); (2) study design (randomized controlled trial vs. controlled trials without random assignment); (3) intervention modality (individual‐ vs. group‐based treatment); (4) presenting problem (individual vs. couple related issues); (5) age; (6) gender; and (7) follow‐up. The last four possible moderators (presenting problem, age, gender, and follow‐up) were examined post hoc by the researchers. They are beyond the three initial moderators included in the research questions, but may have played an important role in outcomes and were, therefore, added during the analysis. Unfortunately, very few studies were couple/family‐based, making it impossible to code for couple/family‐based SFBT as a subgroup of intervention modality.

**Table 2:** Univariate meta‐regression moderator analysis.

Variables	Estimate	SE	*K*/*N*	95% CI	dfs	*p*
Outcome type						
Psychological and mental health	1.237	0.257	34/156	[0.702, 1.773]	19.90	< 0.0001***
Social/relationship	−0.727	0.635	34/156	[−2.090, 0.636]	13.98	0.272
Self‐wellness	−0.349	0.401	34/156	[−1.196, 0.498]	16.78	0.396
General wellness	−0.492	0.588	34/156	[−1.836, 0.852]	8.32	0.426
Personal traits/characteristics	−0.476	0.295	34/156	[−1.846, 0.894]	1.85	0.257
Study design						
Non‐randomized controlled trial	1.305	0.148	34/156	[0.665, 1.945]	1.99	0.013
Randomized controlled trial	−0.356	0.256	34/156	[−1.300, 0.588]	2.39	0.279
Intervention modality						
Group‐based intervention	1.038	0.199	34/156	[0.632, 1.44]	30.94	< 0.001***
Individual‐based intervention	−0.957	0.200	34/156	[−2.895, 0.981]	1.13	0.110
Presenting problem						
Individual	0.938	0.179	34/156	[0.575, 1.30]	27.9	< 0.001***
Couple	0.284	0.864	34/156	[−1.877, 2.45]	5.5	0.754
Age						
Intercept	0.901	0.388	27/138	[0.496, 1.753]	11.34	0.040
Mean of age	0.009	0.119	27/138	[−0.019, 0.036]	8.15	0.479
Gender						
Intercept	1.468	0.574	29/124	[−0.018, 2.955]	4.87	0.052
Percentage of female	−0.004	0.006	29/124	[−0.019, 0.010]	6.69	0.506
Follow‐up						
Test after intervention	0.942	0.211	34/156	[0.511, 1.37]	30.05	< 0.001***
Follow‐up test	0.033	0.0473	34/156	[−0.083, 0.15]	5.78	0.511

*** *p* < 0.001.

Univariate meta‐regression analysis using a single predictor discovered that the outcome type was not a significant moderator. When compared to psychological and mental health outcomes, social/relation outcomes (*b* = −0.727, *p* = 0.272), self‐wellness outcomes (*b* = −0.349, *p* = 0.396), general health outcomes (*b* = −0.492, *p* = 0.426), and personal attributes outcomes (*b* = −0.476, *p* = 0.257) had nonsignificant different treatment effects. In this review, no additional moderators were identified as statistically significant by researchers.

## 5 Discussion

This study examined the outcomes of SFBT with Iranian populations. The overall results from 34 randomized and non‐randomized studies resulted in a treatment effect size of *d* = 0.977, 95% CI [0.591, 1.37], *p* < 0.001, indicating a statistically significant large treatment effect of SFBT interventions. In comparison to non‐SFBT participants, individuals who received SFBT treatment were, on average, 0.977 standard deviations better across all outcome types. These findings support many of the findings from other SFBT meta‐analyses (Franklin et al. 2022; Gong and Hsu 2015; Karababa 2023; Kim 2008; Kim et al. 2015; Park 2014; Stams et al. 2006); however, the large effect size found in this study provides an even more robust indication that SFBT is effective with an Iranian population, whereas many meta‐analyses resulted in small or medium effect sizes. It can be concluded that SFBT is very effective with Iranian individuals.

### 5.1 Outcome Types

One aim of this study was to evaluate if SFBT was more or less effective with differing areas of treatment focus. The results indicated that SFBT was effective at a statistically significant level for two of the five domains, including *psychosocial and mental health* (*g* = 1.24, *p* < 0.001, and *self‐wellness*, (*g* = 0.903, *p* = 0.00274). The overall treatment effect was statistically nonsignificant for *social/relationship outcomes* (*g* = 0.613, *p* = 0.0632), *general wellness outcomes* (*g* = 0.784, *p* = 0.092), and *personal traits/characteristics outcomes* (*g* = 0.825, *p* = 0.049). The statistically significant findings for psychological and mental health outcomes and self‐wellness have been similarly shown in several other studies. Kim (2008) reported a significant small effect result for internalizing behaviors, which are similar to the categories in this study. Similar results have been shown within school populations (e.g., Kim and Franklin 2009), with hospital patients (Gong and Xu 2015), and within social service agencies (e.g., Gingerich and Peterson 2013). The large effect result from the current study strongly supports SFBT's effectiveness with internalizing behaviors like depression and anxiety. These strong results contribute to the overwhelming data that shows that SFBT is effective among the worldwide population.

However, personal traits and characteristic outcomes (e.g., problem‐solving, organization, and cooperativeness) were not statistically significant within this study. These results also support the findings from Kim (2008), that SBFT is less effective with externalizing behaviors. However, more current research from Gong and Hsu (2017) reported that SFBT was trending toward significance within Chinese populations for externalizing behaviors. These mixed results indicate that additional research could be useful in understanding the impact of SFBT on externalizing behaviors. Additional research with the Iranian populations would also add clarity to these initial findings, especially since the results were nearing statistical significance at *p* = 0.049.

It is interesting to note that social/relational outcomes were not statistically significant within this study, given that Franklin et al. (2023) recently reported positive SFBT outcomes for family functioning in their meta‐analysis. Other studies have also concluded that SFBT did not have statistically significant results for couple and family problems (Kim 2008; Neipp and Beyebach 2022). It is important to note that within this study, most participants received treatment within group settings for their issues related to social/relational outcomes, including relationship challenges within the family. This could have been a confounding variable in the effectiveness of SFBT's treatment effect for family/couple‐based problems. Potentially the public settings of group work could have impacted the couples/families' willingness to participate fully. It is also important to note that in some studies included in this meta‐analysis, the researchers included the data from one partner from participants. Having data from both partners would give a more complete picture of the impact that SFBT had on couples and their relationships. In relation to the broader literature of SFBT for couple/family therapy, studies have suggested SFBT being effective for couples and as couple/family‐based interventions (Franklin, Bolton, and Guz 2019, Gingerich and Eisengart 2000). However, with a mixture of evidence available in the literature, additional research about SFBT's effectiveness for issues of couples and relationships, especially with Iranian populations.

The nonsignificant findings for general wellbeing are consistent with Franklin et al.'s (2023) findings. However, these results differ from those found by Zhang et al. (2017) in which a small effect size was discovered for medical and health‐related outcomes, and by Kim and Franklin (2015) in which positive wellbeing outcomes were reported. Given that, relatively few studies considered within this meta‐analysis included general wellbeing as a reported outcome these contradictory results may be due to a small overall sample size. Again, as SFBT continues to grow as a treatment option within Iran and as more research is conducted in Iran it is likely that clarity about this discrepancy will be achieved.

### 5.2 Study Design

A second aim of this study was to determine if the study design would impact the finding outcomes. An overall significant treatment effect was observed among randomized controlled trials (*d* = 0.95, *p* < 0.001). In addition, a result nearing statistical significance (*d* = 1.33, *p* = 0.0104) was observed for non‐randomized controlled trials. These findings support the overall results from most previous SFBT meta‐analyses (Franklin et al. 2023; Kim 2008; Neipp and Beyebach 2022; Zhang et al. 2017). Although these results are encouraging given the relatively recent development of SFBT in Iran, the majority of future investigations should focus on RCTs, similar to the Westernized literature. The increased reliance on RCTs in Iran will strengthen the results and the overall findings. It is likely to bolster confidence for researchers examining Iranian research and could serve to bolster clinical confidence about SFBT within Iran.

### 5.3 Intervention Modality

Similar to other SFBT studies, there was an overall significant treatment effect observed for group‐based SFBT treatment (*d* = 1.04, *p* < 0.001). Group‐based SFBT has been shown to be effective in Bolivia (Pérez Lamadrid and Froerer 2022), Thailand (Lak Thong, Chai Mongkol, and Heng Udom Sap 2017), Taiwan (Hsu, Chen, and Chen 2022), and the United States (Yates, Choi, and Beauchemin 2019) among others countries. For this reason, it is unsurprising that group‐based intervention within Iran would also be effective. In fact, Smock et al. (2008) and Froeschle, Smith, and Ricard (2007) conducted group‐based studies that helped to get SFBT recognized as a promising evidence‐based practice in the United States on the National Registry of Evidence‐Based Programs and Policies through SAMHSA (Kim et al. 2019). This additional, statistically significant, research from Iran shows that group‐based SFBT is useful with various worldwide populations and with differing presenting problems. It is important to note that individual‐based treatment did not show statistically significant results within this meta‐analysis. However, these results are likely due to a very small sample size. Only two of the total 34 studies analyzed included individual‐based treatment. This is a limitation within this study and additional individual‐based treatment should be conducted with Iranian populations before reliable conclusions can be reached about the effectiveness of SFBT with individuals.

Despite the lack of empirical support for individual treatment within Iranian populations, there was a statistically significant result for the outcomes among individual respondents (*d* = 0.931, *p* < 0.001), when compared to couple respondents. Couples and couple‐related issues are commonly studied in Iran; however, results are regularly reported for a single individual rather than from both partners or the dyad as a unit. This practice makes doing dyadic analysis difficult and potentially unreliable. This single‐respondent data collection practice may be for cultural reasons; Iranian men are less represented in the data and could be unwilling to attend therapy or participate in data collection. There may still be a cultural stigma about men seeking therapy in Iran that contributes to these results. Future research should consider male participants and should include dyadic data analysis to overcome this limitation.

In addition to the discussions above, the findings of the study provide important practice implications for marriage and family therapists, especially when providing culturally informed intervention to Iranian populations. Specific clinical tips that are informed by this study include the following: (1) considering the broader cultural context when discussing couples, relationship, and family‐related issues with clients of Iranian backgrounds; (2) being critically aware that while SFBT is a highly promising and research‐validated intervention approach for issues of relationship, more research is needed in this area; and (3) there exists strong evidence supporting SFBT's treatment effect for psychological and mental health outcomes in Iranian populations, making it an evidence‐supported approach.

### 5.4 Limitations of the Study

There are several limitations to this review and meta‐analysis. First, despite our significant efforts to conduct an all‐inclusive search, to secure every article and review every study, it is impossible to guarantee that every Iranian study, published in English, was captured and included in this study. Second, given that much of the SFBT Iranian literature is published in Persian, it is possible that a full review of all Iranian studies (both English and Persian) could have resulted in different findings. We are currently securing and reviewing the Persian language studies from Iran and will compare and contrast the results from that study with the results of this study. However, we felt it valuable to present the current results as a first step to identifying the valuable work coming out of Iran. Third, including both randomized and non‐randomized controlled trials may have impacted the results of this study. We tried to limit the impact of this limitation and consider the impact relatively small because we controlled for study design within the analysis. Fourth, while the use of RVE in meta‐regression alleviated the concern for small sample sizes, including subgroup and moderator analyses, to a certain extent, certain findings would need replication and further investigation when a larger sample size becomes available. Fifth, the authors of this study were unable to account for variations in measurement intervals between studies. As more research becomes available, controlling for this would strengthen the results and findings. Sixth, many of the original studies used waitlist or attention control as comparison groups. Future replication studies with more robust control group comparisons are recommended to strengthen the overall findings of this study. Finally, overall, the studies reviewed had relatively small sample sizes and may have had issues with fidelity. Despite these limitations, to our knowledge, this is the first study to evaluate a relatively sizable clinical trial exclusively focusing on the application of SFBT with an Iranian population. We used a modern meta‐analytical technique that allowed us to include multiple effect sizes in the meta‐regression analysis, which strengthened the statistical power of our analysis as well as enabled moderator analysis.

## 6 Conclusion

This study found support for the overall effectiveness of SFBT with an Iranian population. The results showed an overall large effect size with this population, something that is relatively uncommon to date. Further, it showed a statistically significant effect for psychosocial and mental health presenting concerns as well as for self‐wellness concerns. This study also showed evidence that group‐based SFBT is effective and that analyzing data from individual participants results in effective outcomes. It will be important to add to these results in future research by analyzing the results of studies with Iranian populations published in Persian and by conducting additional RCT studies with more rigorous study designs.

## Supporting information

Supporting information.

Supporting information.
