# Supplementary material for: Meta‐Analysis of Solution‐Focused Brief Therapy Research Conducted in Iran: Does Outcome Type, Intervention Modality, or Delivery Format Make a Difference?
Source: J Marital Fam Ther. 2024 Dec 29;51(1):e12759. doi: 10.1111/jmft.12759 (PMC11683447; doi:10.1111/jmft.12759)
Supplement: Supplementary file 1 — Supporting information. [file JMFT-51-0-s002.docx]

| Risk of Bias for Randomized Controlled Trial | | | | | | |
| --- | --- | --- | --- | --- | --- | --- |
|  | Randomization^1^ | Deviation^2^ | Missingness^3^ | Measurement^4^ | Reporting^5^ | Overall^6^ |
| Javadian et al. 2021 | M | L | L | L | L | L |
| Kafaki et al. 2017 | M | L | L | L | L | L |
| Mortazavi et al. 2020 | L | L | L | L | L | L |
| Khameneh et al. 2014 | M | L | L | L | L | L |
| Namani et al. 2016 | M | L | L | L | L | L |
| Hajian et al. 2013 | M | L | L | L | L | L |
| Javanmiri et al. 2012 | M | H | L | L | L | M |
| Jalali et al. 2017 | M | L | L | L | L | L |
| Ramezani et al. 2016 | L | L | L | L | L | L |
| Mohiti et al. 2022 | L | M | L | L | L | L |
| Farhady et al. 2014 | M | L | L | L | L | L |
| Shahsiah, 2015 | M | H | L | L | L | M |
| Javid et al. 2019 | M | L | L | L | L | L |
| Shirashiani et al. 2017 | M | L | L | L | L | L |
| Rafie et al. 2021 | L | L | L | L | L | L |
| Dinmohammadi et al. 2021 | L | L | L | L | L | L |
| Aminnassb et al. 2018 | L | L | L | L | L | L |
| Khabir et al.2017 | M | H | L | L | L | M |
| Shahbazi er al. 2020 | M | L | L | L | L | L |
| Mirzavand et al. 2016 | M | L | L | L | L | L |
| Hosseinpour et al. 2015 | M | L | L | L | L | L |
| Arkian et al. 2021 | L | M | L | L | L | L |
| Mehrabi et al. 2020 | L | L | L | L | L | L |
| Kivi et al. 2020 | M | M | M | L | L | M |
| Davarniya et al. 2018 | M | L | L | L | L | L |
| Abusaidi et al. 2018 | M | L | L | L | L | L |
| Takalu et al. 2017 | M | L | L | L | L | L |
| Baghernezhad er al. 2019 | L | L | L | L | L | L |
| Abdollahi et al. 2020 | M | L | L | L | L | L |
| Tabatabaei et al. 2019 | M | L | M | L | M | M |
| Bagajan et al. 2016 | L | L | L | L | L | L |
|  |  |  |  |  |  |  |
|  |  |  |  |  |  |  |
|  |  |  |  |  |  |  |
| * L = Low risk of bias (green color); H = High risk of bias (red color); M = Some concerns (Mid-level risk of bias, yellow color)  1. Risk of bias arising from the randomization process  2. Risk of bias due to deviations from the intended interventions (effect of assignment to intervention)  3. Missing outcome data  4. Risk of bias in measurement of the outcome  5. Risk of bias in selection of the reported results  6. Overall risk of bias | | | | | | |

| ROBINS-I for Controlled Trials without Randomization | | | | | | | | |
| --- | --- | --- | --- | --- | --- | --- | --- | --- |
|  | Confounding^1^ | Participant^2^ | Classification^3^ | Deviation^4^ | Missingness^5^ | Measurement^6^ | Reporting^7^ | Overall^8^ |
| Saadatzaade et al. 2012 | L | M | L | L | L | L | L | L |
| Sarvi et al. 2016 | L | H | L | M | L | L | L | L |
| Kargar et al. 2021 | L | M | L | M | L | L | L | L |
|  |  |  |  |  |  |  |  |  |
|  |  |  |  |  |  |  |  |  |
| 1. Bias due to confounding  2. Bias in selection of participants into the study  3. Bias in classification of interventions  4. Bias due to deviations from intended interventions  5. Bias due to missing data  6. Bias in measurement of outcomes  7. Bias in selection of the reported result  8. Overall bias | | | | | | | | |
