# Supplementary material for: Meta‐Analysis of Solution‐Focused Brief Therapy Research Conducted in Iran: Does Outcome Type, Intervention Modality, or Delivery Format Make a Difference?
Source: J Marital Fam Ther. 2024 Dec 29;51(1):e12759. doi: 10.1111/jmft.12759 (PMC11683447; doi:10.1111/jmft.12759)
Supplement: Supplementary file 2 — Supporting information. [file JMFT-51-0-s001.docx]

| **Supplemental Table 1. Study characteristics of included studies** | | | | | | | |
| --- | --- | --- | --- | --- | --- | --- | --- |
| Author | Sample | Demographics | Control* | Population | SFBT Description | Outcome | Measure |
| Javadian et al. 2021 | T= 15  C= 15 | Age = 13.5  %Female = 100%  %White = 0% | WTL | High school female students | **The summary of sessions:**  1^st^ – Introducing the group members to the leader of the group, getting to know the generalities, goals, and effects of the solution-focused perspective.  2^nd^ – Identifying problematic areas, familiarizing with the process of change, not expecting big changes, and emphasizing the change itself.  3^rd^ - Helping the students to identify positive exceptions in life, thereby creating hope and reducing the scope of their problems.  4^th^ - Disrupt the different behavior patterns students have designed using visualization.  5^th^ - Maintaining the change trends, summarizing and rating the changes achieved, targeting for changes, and rating for change toward the desired behavior.  6^th^ – Reviewing past topics, answering questions, and expressing final feelings. | Anxiety; Social skills | SCARED; SSRS |
| Saadatzaade et al. 2012 | T = 22  C = 22 | Age = 16.5  %Female = 0%  %White = NR | WTL | High school students | **Special strategies:**  1 the real pry; 2 speaking in client language; 3 embossing abilities and potentials; 4 commending client; 5 considering changes; 6 focused on here and now; 7 instructing optimize; 8 looking for exceptions to the problem; 9 miraculous question; 10 qualifying the accurate, accessible, and measurable goals; 11 codifying and adjusting the purpose more accurately; 12 assigning homework tasks; 13 looking for strengths or solutions; 14 goal setting.  **Session outline:**  1 Introducing group rules, counseling goals; 2 formulating positive, accurate, and measurable goals; 3 focused on positive exceptions versus reasons; 4 help student to change their thought about academic problems; 5 focused on here and now, and challenge thought that “problems always are in their life and never change”; 6 focused on miraculous questions to disarrange the behavioral patterns of student; 7 diverse topics, response to student’s questions, supporting students to transfer skills to outside environment of school. | Self-regulation;  Academic motivation;  Academic achievement. | SRQ |
| Kafaki et al. 2017 | T = 13  C = 13 | Age = 35  %Female = 76.9%  %White = 0% | TAU | Multiple Sclerosis patients in Sari | **Session goals:**  1^st^ – Making acquaintances; familiarity with distress tolerance and factors affecting it; familiarity with decision making styles and factors affecting them; familiarity with adjustment and factors affecting it; familiarity with short-term solutions focused therapy and factors affecting it.  2^nd^ – Restatement of goals and starting the process with the intervention and activities of subjects.  3^rd^ to 5^th^– Reviewing the goals and receiving feedback about practical actions and determination of new goals or reviewing them. | Distress tolerance; Social adjustment. | DTS; SAS |
| Mortazavi et al. 2020 | T= 62  C= 59 | Age = 26.24  %Female = 100%  %White = 0% | TAU | Pregnant woman | **Topics covered in each session:**  1^st^– Greeting, introducing individuals to each other; helping clients to formulate their goals based on the solution-focused approach and presenting their solutions in the session; homework assignment; feedback.  2^nd^ – Greeting, evaluation of homework; helping clients to realize their abilities, helping them to understand different perspectives on pregnancy and childbirth; homework assignment; feedback.  3^rd^ - Greeting, evaluation of homework; helping clients identify the positive points of pregnancy and childbirth, admiring and praising clients for positive views, and ignoring negative ones; homework and feedback.  4^th^ - Greeting, evaluation of homework; helping clients identify appropriate ways of thinking, feeling, and behaving instead of the current problematic thinking, feeling, and behavior; helping clients find other ways to think, feel, and behave instead of what they have done so far; teaching anxiety reduction and stress management techniques; homework and feedback of the session.  5^th^ - Greeting, evaluation of homework; summarize, conclude and determine whether members have achieved their goals; assess the impact of using the solutions provided to reduce anxiety and childbirth fear; review previous session; pot-test implementation; thanks and appreciation to the members for attending the session. | Pregnancy anxiety; Fear of childbirth(Wijma-delivery expectancy) | ASP; W-DEQ |
| Khameneh et al. 2014 | T= 15  C= 15 | Age = 33  %Female = NR  %White =0% | WTL | Multiple Sclerosis patients | **Structure of intervention sessions:**  1^st^ – Introduction and expression of the aims of sessions, introducing the problem and method of writing reports tor tasks.  2^nd^ – Understanding the approaches for coping with problems from different psychological perspectives.  3^rd^ – Surveying the group members’ problems, using the targeting technique.  4^th^ – Determining situations and solutions, using the technique of exploring exceptions in reducing problems and detecting the moments when the problems and complains are less, using the miraculous questions.  5^th^ – Using the master key technique to do a different task.  6^th^ – Using the master key technique to pay more attention to the pathological behavior and the results of behavior.  7^th^ – Familiarizing with master key technique about writing thoughts, reading them and then burning them, writing negative messages and replacing them with positive messages.  8^th^ – Providing a summary about the subject matters of held sessions and a review about them, answering to all of the questions and uncertainties of group members, implementing the posttest. | Self-confidence; Personal view; Flexibility and adaptability; Organized; Problem-solving; Interpersonal competence; Social relationship; Being active; Resilience. | RQ |
| Namani et al. 2016 | T= 15  C= 15 | Age = 15  %Female = 100%  %White = NR | WTL | Female adolescents | **Protocol of the Intervention:**  1^st^ – Introduction, establishment of communication, determining the framework and rules of sessions, motivating and encouraging the members to focus on solutions instead of problems.  2^nd^ – Clarifying the construct of “sense of coherence”, defining the objectives, stating the issues discussed in the session, training how to review and remold problems.  3^rd^ – Explaining the components of sense of coherence, using a scale of 0 to 10 for prioritizing problems, making contracts and stating possible solutions to problems and highlighting the potential and actual capabilities of students.  4^th^ – Raising the students’ awareness of the benefits of promoting the level of sense of coherence and disadvantages of the failure to do so, finding the exceptions.  5^th^ – Talking about the relationship between the sense of coherence and type of performance in connection with the problems raised by the students, using miracle questions.  6^th^ – Discussion with students about the relationship between the components of sense of coherence with their feeling of peach and health in the face of crises and stressors of their life, stabilizing and consolidating the solutions to problems or reducing them.  7^th^ – Using a scale of 0 to 10 to achieve the goal, helping the members find the methods to think and feel differently.  8^th^ – Summing up the previous matters, conclusion, praising and admiring the students for their success. | Sense of coherence | Flensborg Sense of Coherence Scale (2006) |
| Hajian et al. 2013 | T= 14  C= 14 | Age = NR  %Female = 50%  %White = NR | WTL | Couples who had marital conflicts and less intimacy | **Protocol of the Intervention:**  1^st^ – Communicating and developing good relationship, assessing nature of the problem and level of the marital conflict, finding the chief complaint, defining the problem, re-forming the framework of the participants’ problem, mutual understanding on a given problem, identifying the participants based on their visitor, complainer, and consumer relationships, asking graded questions, giving assignments and feedbacks.  2^nd^ – Revision of the previous session, examining the assignments, determining the objective, exploring the solutions to alleviate the complaint, formulation of solution-problem circles, giving up the old and usual solutions, paying attention to the couples’ resources in dealing with problems, giving assignments and feedbacks.  3^rd^ –Revision of the previous session, examining the assignments, identifying the exceptions, finding a positive story, giving assignments and feedbacks.  4^th^ –Revision of the previous session, examining the assignments, using solution-focused questions, reduction argument, straw man argument, praising the participants, giving assignments and feedbacks.  5^th^ –Revision of the previous session, examining the assignments, using the intensive-term solution-focused couples therapy techniques, giving assignments and feedbacks.  6^th^ –Revision of the previous session, examining the assignments, using graded questions, the extent to which the objectives of the therapy are reached, praising the participants for reaching the objectives. | Psychological intimacy; Spiritual intimacy; Social-recreational intimacy; Sexual intimacy; Emotional intimacy; Intellectual intimacy; Physical intimacy; Relationship intimacy. | Marital intimacy questionnaire |
| Javanmiri et al. 2012 | T= 10  C= 10 | Age = 16.5  %Female = 100%  %White = 0% | WTL | Teenage girls | Treatment group was provided with 8-hour group counseling (8 sessions which took one hour). | Depression | BDI |
| Jalali et al. 2017 | T= 50  C= 50 | Age = 33.85  %Female = 50%  %White = NR | WTL | Married prisoners and their wives | **Record of the Intervention:**  1^st^ – Familiarity between therapist and couples, establishing the fine relationship and therapeutic alliance, describing the rules of therapy sessions. Express the general principles of SFBT. Defining the problem, asking the clients to spell out their problems in a word, and then, converting the word to a sentence, converting the problems to achievable goals, and debating the problems. They were asked to write their goals as an assignment.  2^nd^ – Checking the assignment, setting goals, checking the solutions to resolve problems, and formulating solutions to problems. They were asked to write other expectations from their spouse in specific, tangible, and measurable.  3^rd^ – Summary, checking problems, talking about the future and using exceptional techniques helping couples to recognize the positive exceptions in marital life. Miracle questions were used, helping couples finding a positive story.  4^th^ – Summary and checking assignments, describing the king key technique and using it, using scaling questions to help couples to investigate more solutions and grading the severity of their problems.  5^th^ – Checking assignments, using king key technique, using role playing, using solution-focused questions, using contradictory betting.  6^th^ – Summary, using scaling questions and determining whether the clients have reached treatment goals. The couples are asked to compare his or her performance from the beginning up to this session. Therapists encourage the couples. | Marital satisfaction; Communication; Conflict Resolution; Idealistic distortion. | ECS |
| Ramezani et al. 2016 | T= 25  C= 35 | Age = 26.14  %Female = 100%  %White = NR | ACT; WTL | Pregnant women | **The structure of the counselling:**  1^st^ – Welcome and introduction of group members and a description of the number of sessions and the procedures for following up the participants to complete the questionnaires after delivery; explaining the purpose of counselling sessions; introducing maternity blues and postpartum depression and explaining their causes and symptoms.  2^nd^ – The exact definition of the problem by the client in the form of a sentence and a word; setting objective and operational goals; reviewing the solutions to the problem; providing homework.  3^rd^ – Checking homework; finding exceptions; summing up meeting. | Postpartum blues; Depression | The Persian version of Austin inventory |
| Sarvi et al. 2016 | T= 8  C= 8 | Age = 10.5  %Female = 100%  %White = NR | WTL | Fifth grade primary school female students | **Aim of sessions:**  1^st^ – to familiarize the members of the group with each other.  2^nd^ –to help in targeting and concentrating aims; to apply the best strategies and trained methods.  3^rd^ – to change behavioral patterns resulting from the lack of control over negative emotions; to present the use of solutions to control negative emotions.  4^th^ – disruption of behavioral patterns resulting from the lack of social competence; to practice bravely behaviors.  5^th^ – to summarize and answer questions; to present previous sessions and solutions used by group members; to present 16 questions of miracle for each of the members; to give positive feedback of members to each other; to investigate their own advancement in academic affairs, social proficiency and controlling negative emotions. | Competence; Respect and perseverance; Assertiveness; Self-efficacy. | SEQ-C |
| Mohiti et al. 2022 | T= 51  C= 50 | Age = 23.51  %Female = 100%  %White = 0% | TAU | Nulliparous women | Mothers received a session individually for each parturient by a qualifies psychologist-trained researcher in a labor single room for 45-60 min before the onset of labor pain.  After meeting, miracle questions, scaling questions, the coping questions, and control and exception-finding questions were asked. | Labor pain | SAI; VAS |
| Farhady et al. 2014 | T= 44  C= 44 | Age =  %Female = 50%  %White = 0% | WTL | Couples | **Protocol of the Intervention:**  1^st^ – The objectives are introducing group members to each other and to the group’s leader as well as introducing principles, goals and effects of solution-focused approach and determining the framework and total counseling principals of solution-focused approach.  2^nd^ – To help couples to develop their own targets.  3^rd^ – The objective is to help couples to discover their capabilities and to admire each other in appropriate times and perceive that there might be different interpretations of one single event in family life.  4^th^ – The goal is to help couples recognize positive exceptions in their life with their spouse as in this manner hope is made in them and they can decrease their problems.  5^th^ – The goal is to help couples to eliminate disturbing behavioral patterns by using a miraculous question.  6^th^ –The objective is to help couples take up another way for thinking, feeling, and behavior and help them experience new feelings.  7^th^ – Gathering previous topics, concluding and accomplishing post-test.  8^th^ – Accomplishing follow-up test one month after conducting post-test. | Happiness | OHI |
| Shahsiah, 2015 | T= 50  C= 32 | Age = NR  %Female = 50%  %White = 0% | WTL | Couples | 6 sessions | General marital conflict; Reduced cooperation; Reduced sexual intercourse; Increased child support Increased personal relationships with relatives; Reduced family relationship with the spouse’s relatives; Separating finances; Reduction of effective communication. | Marital conflict questionnaire |
| Javid et al. 2019 | T= 20  C= 20 | Age = 21  %Female = 100%  %White = 0% | WTL | Midwifery students | **Protocol of the Intervention:**  1^st^ – Initiation of appropriate and trustful relationship, stating the goals of the sessions, trust making, familiarizing and introduction, stating the counseling procedures and regulations of the group, familiarity of the students with the concepts of mental health, talking about the future, and giving homework.  2^nd^ – Discussing about the homework, imaging the problems and proposing solutions for the problem, making participants committed and hopeful to solve problems, identifying and solving the resistance of the participants, using the technique of comparative questions, investigating eliminating solutions, complaints, formulating the trend of problem solving, highlighting and clarifying their capabilities and abilities in problem solving, encouraging the members to investigate the solutions, giving homework.  3^rd^ – Checking homework, talking about future, making students familiar with problem exceptions, using exception questions, empowering their capabilities and abilities, summing up the information, and implementing the miracle questions.  4^th^ – Checking homework, helping the participants to identify other ways of thinking, of feeling and of behaving, encouraging the participants to check the solutions, emphasizing on their capabilities and abilities in the field of social performance, using comparative questions, using the master key technique, and giving homework.  5^th^ – A summary of the previous session, checking the homework of the previous session, summing up and conclusion, and determining whether the students have achieved the counseling goals or not. Stating that the solution of problems is in people, and that they can learn and use them. Holding post-test. | Mental health; Physical symptoms; Anxiety; Social performance; Depression. | GHQ |
| Shirashiani et al. 2017 | T= 12  C= 12 | Age = NR  %Female = 100%  %White = 0% | WTL | Incompatible Iranian women | **Overall purpose of sessions:**  1^st^ – Introduction of therapeutic approaches.  2^nd^ – Externalization and familiarity with the basic principles of solution-focused counseling.  3^rd^ – Attitude towards the problem.  4^th^ – Dominance and rule over the individual  5^th^ – Members’ familiarity with exceptions of the problem.  6^th^ – Techniques to deal with negative emotions towards stressful relationships of the past.  7^th^ – Rewriting the life narrative from past to present.  8^th^ – Summing up the sessions and conclusion. | Dyadic satisfaction; Dyadic cohesion; Dyadic consensus; Affectional expression. | Spinner Marital Adjustment Scale |
| Rafie et al. 2021 | T= 28  C= 28 | Age = 32.5  %Female = 100%  %White = 0% | WTL | Women with gestational diabetes | **Content of sessions:**  1^st^ – Introduction, getting a brief history of the patients’ lives, scaling the stress and anxiety levels, anxiety and group discussion about pregnancy and diabetes- assigning homework.  2^nd^ – Reviewing the homework; the stage of hoped-for outcomes: the "preferred future"; counseling about  what the patients expect of such training program and hope to achieve; each patient explains her problem. Assigning homework.  3^rd^ – Reviewing the homework; helping the patients to set their goals based on the solution-focused approach; using the master key technique and scaling questions. Participants were helped to realize their abilities and capabilities and be able to admire each other. Assigning homework.  4^th^ – Reviewing the homework; determining expectations and needs with the help of authorities; having the sense of responsibility and hope to resolve the problems by different solutions, identifying the causes for clients’ resistance to solve the problems, highlighting their abilities to  solve the problems. Assigning homework.  5^th^ – Reviewing the homework; explaining that there are exceptions for any problem, using the miracle question by visualizing a person in a bad or a good situation in order to enable the members to understand that bad or good moments are the result of the person’s thoughts and behaviors and help the people to understand the problems or perceived barriers against changes.  6^th^ – Helping the patients to use other ways of thinking about a subject and describing their emotions and behaviors about that way of thinking; using comparative questions; thus, the counselor ensures that the patients moved forward towards changes and help members to find other ways of thinking, feeling, and behaving instead of what they are doing, and experience new emotions. At the end, making conclusion and summary of the content. | Stress; Depression; Anxiety; Coping styles-solution; Coping styles-emotion; Coping styles-avoidance. | DASS-21; CISS |
| Dinmohammadi et al. 2021 | T= 41  C= 41 | Age = 27.55  %Female = 100%  %White = 0% | WTL | Pregnant women | **The themes in sessions:**  1^st^ – Highlighting general principles of solution-focused counseling and providing proper definitions of problems to clients.  2^nd^ – The familiarity of participants with the concept of quality of life and solution-focused approach.  3^rd^ – Clients learnt that there are different interpretations for an event and that they can develop the best interpretation in their minds.  4^th^ – Clients were encouraged to discover exceptional opportunities of living as a couple.  5^th^ – With the help of miracle questions, participants were able to recognize their destructive behavior patterns.  6^th^ – A conclusion was made from the whole previous sessions to help the clients replace and experience their former thoughts and behaviors with the new ones. | Negotiation; Psychological abuse; Minor physical violence; Severe physical violence; Sexual abuse; Quality of life; Vitality; Mental health; General health; Bodily pain; Physical functioning; Role limitation-physical health; Role limitation-emotional health; Social functioning. | CTS-2; SF-36 |
| Aminnassb et al. 2018 | T= 15  C= 15 | Age = 35  %Female = 100%  %White = 0% | TAU | Patient with breast cancer | **Protocol of the Intervention:**  1^st^ –Explain the principles of the therapeutic sessions; introduction and expression of the aims of sessions; introducing the problem and method of writing reports for tasks. Setting the goals in a positive, specific, tangible, and measurable way.  2^nd^ – Understanding the approaches for coping with problems from different psychological perspectives.  3^rd^ – Omission of the disruptive behavior and cognitive patterns using the miracle question, using the targeting technique.  4^th^ – Determining situations and solutions, using the technique of exploring exceptions in reducing problems and detecting the moments when the problems and complaints are less for understanding the existence of positive exceptions in life, increasing hope, and reducing the level of problems.  5^th^ – Realizing own abilities when needed. Using the master key technique: doing a different task.  6^th^ – Learning new ways of thinking, feeling, acting and  behaving, and experiencing new feelings using the very significant word “instead.” Using the master key technique: paying more attention to the pathological cognitive and behavior and the results of behavior and cognitive  7^th^ – Clear understanding of the participants, of changes made in their lives by themselves, and realization of the personal skills they have used in the process. Familiarizing with master key technique: writing thoughts, reading them and then burning them, writing negative messages and replacing them with positive messages.  8^th^ – Providing a summary, answering to all the questions and uncertainties, implementing the posttest. | Depression; Perceived Stress | PSS; CES-D |
| Bagajan et al. 2016 | T= 15  C= 15 | Age = NR  %Female = 100%  %White = 0% | WTL | Family supervisor women | **The treatment plan:**  1^st^ – Group member familiarity and psychologist to each other and making a relationship to make a relation without bias and absolute acceptation of clients.  2^nd^ – Introduction session affairs and program, speaking about problem and then expressing problem by clients presenting technic of exceptional cases which is based on client’s abilities and capabilities and miracle question technic about someone who claim that they have not experienced exceptional case.  3^rd^ – Expressing briefly, what was presented in the previous session by members, persuading members’ duties, and suitable feedback to correct duties and remove problems of incorrect duties by clients and group members. Changing goals to smaller solutions, each of these smaller solutions is changed to one goal and presenting four traits for choosing solutions.  4^th^ – Helping to members to remove errors in order to reach rational, clear, objective, executable solution and strengthen clients’ effort by other members, asking about what are small next steps to reach the perfect goal.  5^th^ – Correcting errors – expressing solutions which they have done to reach their goals and its effect on their problems; helping group members in recognizing when they have been improved sufficiently and how they try to repeat if after ending therapy session, directing generalization of sessions achievements to other situations, asking | Psychological wellbeing | RSPWB |
| Khabir et al.2017 | T= 12  C= 12 | Age = 36  %Female = 50%  %White = NR | WTL | Parents of children with cancer | NR | Intrusion; Avoidance; Hyperarousal | Impact of Events Scale |
| Shahbazi er al. 2020 | T= 17  C= 20 | Age = 24  %Female = 50%  %White = 0% | WTL | Probative university students | **Supporting sources:**  Walther and Peller, Mudd, Lipchick and Nelson and Thomas.  **Goal for each session:**  1^st^ – Establishing therapeutic relationship, making individuals familiar with studied subject, execution of pretest.  2^nd^ – Conversion of problem into accessible goals.  3^rd^ – Analysis on solutions to remove complains.  4^th^ – Analysis on level of commitment and hope of care-seekers.  5^th^ – Finding a positive story to analyze exceptions.  6^th^ – Training of finding a solution in different situations using magic question.  7^th^ – Finding different solutions for emotion, thought and behavior.  8^th^ – Analysis on constructive changes and stabilizing and consolidation of them.  9^th^ – Conclusion of sessions, execution of posttest. | Novelty seeking; Harm avoidance; Reward dependency; Persistence; Cooperativeness; Self-directedness; Self-transcendence; Temperament/Character dimensions | TCI-125 |
| Mirzavand et al. 2016 | T= 20  C= 20 | Age = 27  %Female = 50%  %White = 0% | WTL | Couples who applied for divorce | **Protocol of the Intervention:**  1^st^ – Introduction, explaining objectives, process and laws, and performing the technique to bypass the objectives of the members of the group.  2^nd^ – The explanation of fundamental principles of solution-focused therapy. Commenting on how the members of the group should discuss the issues and reach an agreement, decentralization of negative items, and emphasis on positive ones.  3^rd^ – Using grading scale of evaluation of the level of commitment and hope, and solving their problem, and identifying resistance of the members using questions of how, when, what and where.  4^th^ – Encouraging members to express their problems, taking care of their children, communicating with relatives and talking about their solutions, encouraging group members to talk about useful solutions.  5^th^ – Asking questions and talking about a ‘miracle question’, encouraging members to solve their problem, and discussing their strengths.  6^th^ – Asking questions and answering a ‘miracle question’ and using alternative ideas of “instead of” regarding their replies in the group.  7^th^ – Using the term ‘appropriate behavior and proper feelings’ instead of using problematic feelings and thoughts and behavior, and using technique of 180-degree turning, asking the members to mention at least two positive features of their spouses, thanking them.  8^th^ – Summarizing the meetings and asking members’ comments on previous sessions, performing the posttest and thanking members for cooperation and participation in meetings. | Marital burnout; Physical wearing; Emotional wearing; Psychological wearing; Marital burnout | Pine’s couple burnout scale |
| Hosseinpour et al. 2015 | T= 15  C= 15 | Age = 16.5  %Female = 100%  %White = 0% | WTL | High school female students | **Protocol of sessions:**  1^st^ –Interactive understanding between the researcher and the participants. Being familiar with the concept of adjustment and the factors affecting brief therapy. Being familiar with solutions-focused brief therapy sessions.  2^nd^ – Restating programs and goals of solution-focused brief therapy and starting and targeting therapy along with subjects’ intervention and activity programs.  3^rd^ to 5^th^–Review and monitor the plan and the objectives of the previous session, gather feedback regarding treatment, gather feedback regarding acted, set new goals or revising them. | Home adjustment; Health adjustment; Emotional adjustment; Social adjustment | Four subscales of the questionnaire introduced by Bell in 1961: home adjustment in the house, health adjustment, social adjustment, and emotional adjustment. |
| Arkian et al. 2021 | T=  C= | Age = 14.3  %Female = 0%  %White = 0% | ACT; WTL | High school students | Ten subjects participated in the SFBT intervention group and were subjected to Gutterman advanced techniques for Solution-Focused Counseling Protocol in eight 90-minute group sessions. | Autonomy; Environmental mastery; Personal growth; Positive relations with others; Purpose in life; Self-acceptance | RSPWB-SF |
| Mehrabi et al. 2020 | T= 37  C= 38 | Age = 55.8  %Female = 100%  %White = 0% | TAU | Postmenopausal women suffering from empty nest syndrome | **The contents of the sessions**:  1^st^ –Introduction, problem determination, determining the objectives of the consultation sessions.  2^nd^ – Defining and highlighting exceptions, using graded scale questions.  3^rd^ – Reviewing assignments and summaries from the previous session, using miraculous questions and talking about the future, finding a positive story in life, using solution-focused questions, highlighting abilities and talents.  4^th^ – Reviewing the assignments of the previous week, using the important word "instead", using the master key technique, using graded questions.  5^th^– Reviewing the assignments of the previous session, summarizing the topics, taking the post-test.  6^th^ – Using the experiences of one of the subjects with empty nest syndrome. | Happiness; Life satisfaction; Self-esteem; Actual well-being; Contentment; Positive mood. | OHI |
| Kargar et al. 2021 | T= 30  C= 30 | Age = 39.3  %Female = 100%  %White = 0% | ACT | Women with high body mass index | **The contents of the sessions**:  1^st^ – Fostering communication and familiarity, provision of the goals, rules, and procedures of the sessions, instillation of hope for change in the participants.  2^nd^ – Familiarity with the basic principles of solution-focused consulting and its application, examining the active acceptance of responsibility in a relationship, formulating problem-solving circles.  3^rd^ – Identifying the solutions for different problems of participants, familiarizing the participants with useful solutions, reinforcing them, and abandoning inappropriate solutions, finding a positive story.  4^th^ – Commitment and instillation of hope in participants for solving problems, identifying and overcoming participants' resistance, talking about the future and measures, using the technique of scale questions.  5^th^– Members' familiarization with exceptions, strengthening and highlighting exceptions, participants' realization of their capabilities and potentials.  6^th^ – Helping participants identify other methods of thinking, feeling, and behaving instead of the current problematic thinking, feeling, and behavior, helping participants to admire each other instead of criticizing, and performing the master key technique.  7^th^ – Helping participants to imagine their favorite future and a better world, helping participants to get out of the problem scope, identifying objective and desirable changes, using the technique of predictive task design.  8^th^ – Summarizing the sessions and arriving at a conclusion, using graded questions, thanking and appreciating the members for participating in the meeting. | Desire to have sex; Sexual attitude; Quality of sexual life; Sexual compatibility; Sexual satisfaction | Personal and midwifery information questionnaire; Larson’s Sexual Satisfaction Questionnaire. |
| Kivi et al. 2020 | T= 15  C= 15 | Age = NR  %Female = NR  %White = 0% | ACT | People with over anxiety disorder | **Supporting sources:**  The pattern for intervention based on the Zimmerman, Prest, Wetzel.  **The themes of the sessions:**  1^st^ – Referral, creating relationships, determining the framework and rules of the consultation sessions, a comprehensive explanation of postmodern and solution-focused schools’ pre-trial execution.  2^nd^ – Determining the goals, discussing the issues review training and re-challenge the problems.  3^rd^ – Using the 0-10 scale to prioritize problems and create contracts and express possible solutions to problems.  4^th^ – Finding the exceptions.  5^th^– Using the miracle questions.  6^th^ – Establishing and consolidating solutions to problems or ways to reduce them.  7^th^ – Using the 0-10 scale to find out what your goals are and teaching the keystone to problems with examples of problems with their own lower priority.  8^th^ – Summarizing the past contents and conclusions. | Anxiety | BAI |
| Davarniya et al. 2018 | T= 15  C= 15 | Age = 38.53  %Female = 100%  %White = 0% | WTL | Women who suffering from couple burnout | **Supporting sources:**  The protocol of treatment sessions was developed based on the couple therapy model of Zimmerman, Prest, and Wetzel, and group therapy model of Nazari.  **The aims and tasks of the therapy sessions:**  1^st^ – Performing pretest; getting acquaintance among the group members and the therapist; describing the group rules, determining frameworks, and introducing general principles of therapy. Participants were expected to write down their purpose for participation in sessions and present them to the group in the following session.  2^nd^ – Helping the participants to develop their own purpose in a positive, clear, tangible and measurable way. Participants were asked to write down additional purpose and expectations they have for themselves, spouse, and their lives in a positive, exact, tangible and measurable way and bring them for the next session.  3^rd^ – Helping the clients to learn that there are different interpretations for the same event in the family, and to learn to change their interpretation of the problems in a more helpful way. Helping them to know their own resources and capabilities and to learn to admire one another. The group members were asked to refrain from criticizing their spouse in any circumstance, and instead, admire and compliment any positive action done by their spouse and report them all in the following session.  4^th^ – Helping the participants to identify positive exceptions in their marital life, accordingly, find hope in their lives, and be able to reduce their scope of problems. The participants were asked to think more over the proposed questions, identify positive exceptional moments of their lives and report back.  5^th^–Eliminating the disturbed behavioral patterns by using miracle questions. The participants were asked to think more over the proposed questions at home and bring their questions in the following session.  6^th^ – Helping the members to find alternative ways of thinking, feeling, and behaving other than what they currently do, and experience new feelings by using the important term “instead”. The participants were asked to toss a coin in a specific hour every day. The winner can complain from his/her spouse for 10 minutes, after which, the other spouse can complain for 10 minutes. They were asked to report the complains’ result in the following session.  7^th^ –Summarization, conclusion, and exploring if the members have gained their purposes or not? Performing the posttest. Coordination for holding the follow-up session in the following month. | Burnout | Pine’s Couple Burnout Measure |
| Abusaidi et al. 2018 | T= 12  C= 12 | Age = 34  %Female = 100%  %White = 0% | WTL | Married women | **The explanation of sessions:**  1^st^ – Becoming familiar members with each other and with therapist, stating rules group, determining the frameworks and expressing the general principles of short solution-focused therapy. Participants were required for the next session, write their purpose of their participation in the meetings and bring it to the group.  2^nd^ – Helping participants to development their aims positively, certainly, tangible and measurable and bring it to the group.  3^rd^ – Helping participants to understand that there are different interpretations of an event in the family and be able to change their considerations of the problems occurred in a better way. Helping them to realize their own capabilities and resources and admire each other. Participants were required during the week not all complain their husbands, instead admire and appreciate any positive activity form their husbands and bring it to the group.  4^th^ – Help participants to recognize the positive exceptions in their lives with their husbands. And on this basis, the hopefulness will be increased, and they will be able to reduce their problems. Participants were required to think more about the raised questions and recognize the positive moments in their lives and bring it to the group.  5^th^– Eliminate the patterns of disruptive behaviors that resulted by participants using the question of miraculous. Participants were required to think about the raised questions at homes and bring the responses for the next sessions.  6^th^ – Helping members to find other ways of thinking, feeling and behavior rather than what they are doing now and experience new feelings using the important work instead of. Participants were required to identify the rate of improvements in their relations with a scale ranging from 0 to 10 and then bring the result for the nest session.  7^th^ – Summarizing and conclusion and determining whether members have achieved their goals? | Mutual constructive; men demand-withdraw; woman demand-withdraw; men and woman demand-withdraw; mutual avoidance | CPQ |
| Takalu et al. 2017 | T= 15  C= 15 | Age = 48.5  %Female = 53.3%  %White = 0% | WTL | Patient with multiple sclerosis | **The content of sessions:**  1^st^ – Introducing and familiarizing the therapist with group members and encouraging caregivers to share their concerns and problems.  2^nd^ – Questions were asked to explore the concerns and problems of each caregiver.  3^rd^ – Defined and described the goals of caregivers using the miracle questions and scaling questions in a bid to identify potential exceptions.  4^th^ – Ask questions about caregivers’ strengths and resources; propel sessions by finding exception questions to encourage caregivers; request caregivers to write down problems, goals and exceptions that have been identified during past meetings and are ready to be presented at a future meeting.  5^th^– Review and discuss the assignments of the previous meeting in the group, requesting caregivers to review the strengths and resources, and share the proposed matching strategies with other members of the group.  6^th^ – Request caregivers to report progress during the last 5 sessions and discuss as well as hold discussions with other members of the group regarding their progress and plans in this area. During these sessions, if the therapist feels there is progress in people, he has to use the techniques to compliment statements. | Depression; Anxiety; Stress | DASS-21 |
| Baghernezhad er al. 2019 | T= 15  C= 15 | Age = 35.7  %Female = 100%  %White = 0% | ACT; WTL | Patient with breast cancer | **The objectives of sessions**:  1^st^ – Holding therapeutic sessions, familiarizing participants with the research subject, and running a pretest.  2^nd^ – Transforming a problem to an accessible objective.  3^rd^ – An investigation into solutions to deal with a complaint.  4^th^ – An investigation into patients’ commitment and expectation.  5^th^ – Finding an effectual story accompanied by an investigation into exceptions.  6^th^ – Training solution-making methods on different occasions, using a miracle question.  7^th^ – Finding different kinds of methods to utter feelings, thoughts, and behaviors.  8^th^ – An investigation into effective changes to stabilize them.  9^th^ – Closing sessions, running a posttest. | Pain catastrophizing; Psychological well-being | Pain Catastrophizing Scale; Psychological Well-being Questionnaire |
| Abdollahi et al. 2020 | T= 15  C= 15 | Age = 57.73  %Female = 46.7%  %White = 0% | WTL | Patients with cardiovascular diseases | **Contents of sessions:**  1^st^ –Introducing and starting communication, presenting a brief description of solution-focused therapy, and especially emphasizing their ability to solve a problem.  2^nd^ – Focusing on the goal and mentioning the problem, firstly checking the assignment of the previous session, and then asking members to state their goal for participating in the group.  3^rd^ – Focusing on the solution, checking precious session assignments, and asking members to understand their ability to find the problem and stating what they will do if they take a small step to solve their problem?  4^th^ – Presenting a summary, every member should make a list of solutions for him/herself using other member’s experiences.  5^th^ –Presenting a summary, stating individuals’ problems with communication and social function, and receiving other members’ strategies.  6^th^ – Using the critical work of “instead”; checking the assignments and expressing the goals of the current session.  7^th^ – Grading questions, checking the assignments of the previous session, and then using graded questions to better understand the emotions and wishes of the participants and their progress.  8^th^ – Asking group members to continuously discuss their progress and increase the ability to change in him/herself as the solution to the problems is hidden inside themselves and they can solve their problems and then conducting posttest. | Physical symptoms; Anxiety; Social dysfunction; Depression; Mental health; Hopefulness | Snyder’s Hopefulness Questionnaire; Public Health Questionnaire |
| Tabatabaei et al. 2019 | T= 20  C= 20 | Age = NR  %Female = NR  %White = 0% | WTL | Patient with multiple sclerosis | **Protocol of the intervention**  1^st^ –Welcoming, the introduction of group members to the therapist and each other, setting a relationship among group members, explanation of targets and rules of the group, general description of the therapeutic approach, and finally devoting time to each individual to share behaviors, thought patterns, environments, and relationships that might lead to unnecessary anxiety.  2^nd^ –Sharing the participants’ personality characteristics with the group, developing a list of strong points and sharing with the members.  3^rd^ – Checking assignments and reviewing the two previous sessions, group investigation of the ways to achieve stressful targets outside the therapeutic group.  4^th^ –Checking assignments and reviewing the three previous sessions, setting goals, and discussion of achieved goals during the time.  5^th^ –Reviewing and discussing assignments of the previous session, grading anxiety level and general well-being, devoting time to each individual to share behaviors, thought patterns, environments, and relationships that might lead to his/her solace.  6^th^ –Devoting time to review the most important shared moments during the previous five sessions, thinking about information and training items, and giving opinions regarding the plan by members and the therapist. | Generalized anxiety disorder | GAD |
| * For control groups: ACT = active comparator; TAU = treatment as usual or standard care; WTL = waitlist or attention control | | | | | | | |
| **SCARED =** The Screen for Child Anxiety Related Emotional Disorder; **SSRS =** The Social Skills Rating System; **SRQ** = The Self-Regulation Questionnaire; **DTS** = Distress Tolerance Scale; **SAS** = Social Adjustment Scale; **ASP** = The Anxiety Scale for Pregnancy; **W-DEQ** = The Wijma Delivery Expectancy Questionnaire; **RQ** = Resilience Questionnaire; **BDI** = Beck’s depression questionnaire; **ECS** = Enriches Couple Scale; **SEQ-C** = Self-Efficacy Questionnaire-Children; **SAI** = Spielberger anxiety inventory; **VAS** = Visual Analog Scale; **OHI** = The Revised Version of Oxford Happiness Inventory; **GHQ** = The General Health Questionnaire; **DASS-21** = Depression, Anxiety and Stress Scale; **CISS** = Coping inventory for stressful situations intervention; **CTS-2** = Conflict Tactics Scale; **SF-36** = Short Form Health Survey; **PSS** = Cohen’s Perceived Stress Scale; **CES-D** = Center Epidemiological Studies Depression Scale; **RSPWB** = Ryff Scale Psychological Wellbeing;**TCI-125** = Cloninger’s Temperament and Character Inventory; **RSPWB-SF** = Ryff Scale Psychological Wellbeing-Short Form; **BAI** = Beck Anxiety Inventory; **CPQ** = The communication Patterns Questionnaire; **GAD** = Generalized Anxiety Disorder Inventory. | | | | | | | |
